# A Combination of Metabolomics and Machine Learning Results in the Identification of a New Cyst Nematode Hatching Factor

**DOI:** 10.3390/metabo12060551

**Published:** 2022-06-16

**Authors:** Lieke E. Vlaar, Benjamin Thiombiano, Davar Abedini, Mario Schilder, Yuting Yang, Lemeng Dong

**Affiliations:** Plant Hormone Biology Group, Green Life Sciences Cluster, Swammerdam Institute for Life Sciences, University of Amsterdam, Science Park 904, 1098 XH Amsterdam, The Netherlands; l.e.vlaar@uva.nl (L.E.V.); b.thiombiano@uva.nl (B.T.); d.abedini@uva.nl (D.A.); m.l.schilder@uva.nl (M.S.); y.yang@uva.nl (Y.Y.)

**Keywords:** cyst nematode, hatching stimulant, solanoeclepin A, metabolomics, random forest, Pearson’s correlation, *Globodera*

## Abstract

Potato Cyst Nematodes (PCNs) are an economically important pest for potato growers. A crucial event in the life cycle of the nematode is hatching, after which the juvenile will move toward the host root and infect it. The hatching of PCNs is induced by known and unknown compounds in the root exudates of host plant species, called hatching factors (HFs, induce hatching independently), such as solanoeclepin A (solA), or hatching stimulants (HSs, enhance hatching activity of HFs). Unraveling the identity of unknown HSs and HFs and their natural variation is important for the selection of cultivars that produce low amounts of HFs and HSs, thus contributing to more sustainable agriculture. In this study, we used a new approach aimed at the identification of new HFs and HSs for PCNs in potato. Hereto, root exudates of a series of different potato cultivars were analyzed for their PCN hatch-inducing activity and their solA content. The exudates were also analyzed using untargeted metabolomics, and subsequently the data were integrated using machine learning, specifically random forest feature selection, and Pearson’s correlation testing. As expected, solA highly correlates with hatching. Furthermore, this resulted in the discovery of a number of metabolite features present in the root exudate that correlate with hatching and solA content, and one of these is a compound of *m*/*z* 526.18 that predicts hatching even better than solA with both data methods. This compound’s involvement in hatch stimulation was confirmed by the fractionation of three representative root exudates and hatching assays with the resulting fractions. Moreover, the compound shares mass fragmentation similarity with solA, and we therefore assume it has a similar structure. With this work, we show that potato likely produces a solA analogue, and we contribute to unraveling the hatch-inducing cocktail exuded by plant roots.

## 1. Introduction

Potato (*Solanum tuberosum*) is an important staple food and a major source of starch. Its production has increased tremendously in the past decades, making it one of the crops that is feeding the growing world population, especially in developing countries [1]. With the adaptation of potato to cultivation in a range of environments, combined with the variation available in the form of genetic resources, there is potential to further optimize potato production and, for example, more effectively combat pests [1].

Plant-parasitic nematodes are one of the most harmful pests in agriculture, since they result in losses of up to USD 80 billion annually [2]. Among them are the cyst nematodes, comprising the genera *Globodera* and *Heterodera*, together the second most important parasitic nematode species economically [3]. Their host range is highly specific, as opposed to other species such as the root-knot nematode. For example, the potato cyst nematode (PCN, *Globodera pallida* and *Globodera rostochiensis*) is the only parasites species of the Solanaceae.

The life cycle of cyst nematodes is divided into four larval stages and one adult stage, separated by moults. Juvenile stage 2 (J2) larvae use chemotaxis to locate a suitable host, penetrate the host root, and subsequently establish a feeding structure, the syncytium. After moulting into J3, J4, and adults, the female stays sedentary, whereas the male is migratory and finds the female to fertilize her eggs. During egg production, the female body swells and protrudes from the root epidermis. Upon death, the female body will dry and harden and thus turns into a cyst that remains in the soil when the crop is harvested. In the cyst, the embryos in the eggs moult from J1 into J2 and then arrest developmentally.

Cysts with J2 can stay dormant in the soil for many years, until they perceive a cue from their host [4]. This is especially true for *Globodera* spp. that rely almost exclusively on host root exudate for hatching, while other cyst nematodes, such as the beet cyst nematode (*Heterodera schachtii*), may also hatch through rehydration only [5]. Root exudates contain hatching factors (HFs), which induce hatching independently, and hatching stimulants (HSs), which by themselves are inactive but enhance the hatching activity of HFs [6]. For example, Byrne et al. (1998) showed that the hatch-inducing activity of glycoalkaloids, such as α-chaconine and α-solanine, which are well-known HFs, is increased when they are combined with certain potato root exudate fractions that contain HSs. To date, the strongest HF for PCNs that has been identified is solanoeclepin A (solA) [7], which can induce up to 80% hatching in *G. rostochiensis* in nanomolar concentrations [8]. SolA has been identified in the root exudate of a range of solanaceous species, including potato and tomato [7,9]. In kidney bean, three eclepins have been identified: glycinoeclepin A, B, and C [10,11]. These eclepins induce hatching of the Soybean Cyst Nematode (SCN) of the genus *Heterodera*, but not to the same extent. Whereas glycinoeclepin A (glyA) is active at 10^−11^ to 10^−12^ g/mL, glyB and glyC are active only at higher concentrations, around 10^−8^ to 10^−9^ g/mL [10,11]. The variation in the glycinoeclepin structure suggests that more solanoeclepins might exist and that their hatch-inducing activity can vary. In addition to solA, some weaker HFs have been identified, such as α-chaconine, α-solanine, solasodine, and solanidine [12,13]. Apart from hatch-inducing compounds, hatch inhibitors (HIs) are also known. They are, for example, present in the root exudate of young plants preventing egg hatching before enough root material is available for a successful infection [6]. Once the ratio HF:HI is high enough, hatching will be induced.

Although PCNs can infect a range of species of the Solanaceae, potato is economically most affected. Little is known about the natural variation in the hatch induction rate of exudates and solA production in potato, which leads to unpredictable levels of PCN infections. In the present study, we analyzed the solA content in the root exudates of 51 potato cultivars, and, on a selection of these cultivars, we also conducted hatching assays and an untargeted metabolomics analysis. This study aimed to gain more information on the hatch-inducing cocktail of root exudates, which allows for the selection of cultivars with low amounts of HFs and HSs. We found that solA alone does not completely predict hatch-inducing activity of potato root exudates, and therefore we expected to find more, hitherto unknown HFs and/or HSs. Through machine learning with random forest (RF) feature selection and Pearson’s correlation analysis, we identified several metabolic features that predict PCN hatching and correlate with solA. Furthermore, we chromatographically fractionated three exudates with different solA content and tested the resulting fractions with hatching assays. Fractions that induced hatching were once more analyzed on LC-MS. This study identified a new potential HF for PCNs, which will advance our understanding toward plant–nematode interaction at the preparasitic stage and possibly offer alternative solutions to develop resistant crops to these detrimental pests.

## 2. Results

### 2.1. Natural Variation in the SolA Content of Root Exudates Obtained from Potato Cultivars Is Associated with Differences in Hatching Activity

**Figure 1:**
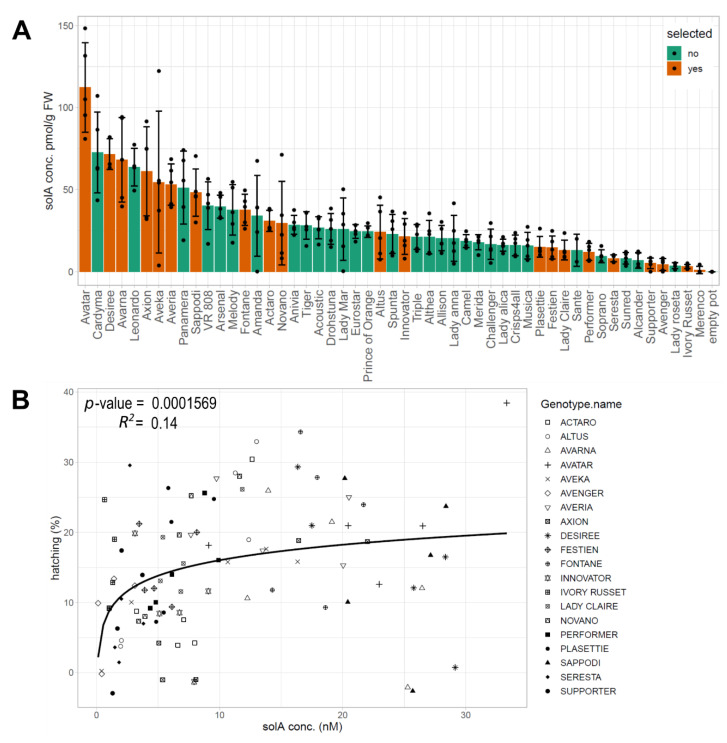
(**A**): solA concentrations in pmol/g FW of 51 commercial potato cultivars measured by UHPLC-MS/MS (solA_MRM). Colors indicate whether the species was selected for further experiments: red yes, green no. (**B**): solA concentrations of selected cultivars from A plotted against average hatching percentage. A logarithmic relationship was detected with *R*^2^ value of 0.14 and a significant *p*-value.

Solanoeclepin A content was analyzed in the root exudates of 51 potato cultivars (Figure 1A) and differed greatly among the cultivars, ranging from 1.1 (Merenco) to 112.3 pmol/g FW (Avatar). SolA was not detected in bulk soil (Figure 1, empty pot). Twenty cultivars with representative solA content were selected for further experiments (Figure 1A). The hatching activities of root exudates of these cultivars were evaluated through a hatching assay with *G. rostochiensis* eggs. All root exudates induced more hatching than the flowthrough of pots with only soil. The hatching correlated positively with SolA content according to a logarithmic relationship, similar to what was observed previously for (wild) tomato (*S. lycopersicum, S. sisymbriifolium, S. habrochaites, S. pennellii,* and *S. pimpinellifolium*) species [9]. Although the *p*-value shows the relationship is significant (>0.001), the R^2^ is low (0.14) (Figure 1B). This low correlation coefficient suggests that there are more compounds present in the root exudates than only solA that influence the hatching activity, such as HSs, HIs, and other HFs [6].

### 2.2. Root Exudates Contain SolA and SolA-Like Compounds That Predict Hatching

In order to say more about the possible role of other compounds in hatching, the root exudates of the twenty selected potato cultivars were also analyzed by LC-ESI-QTOF-MS. PCA was used to visualize the differences in the root exudate metabolome of the potato cultivars (Figure S1). Although variability between replicates was high for some of the cultivars, PC1 and PC2 together explained 55.29% and 73.11% of the total variance of features for the positive and negative modes, respectively. The exudate from Fontane was clearly chemically different from the exudate collected from Axion, for example.

**Figure 2:**
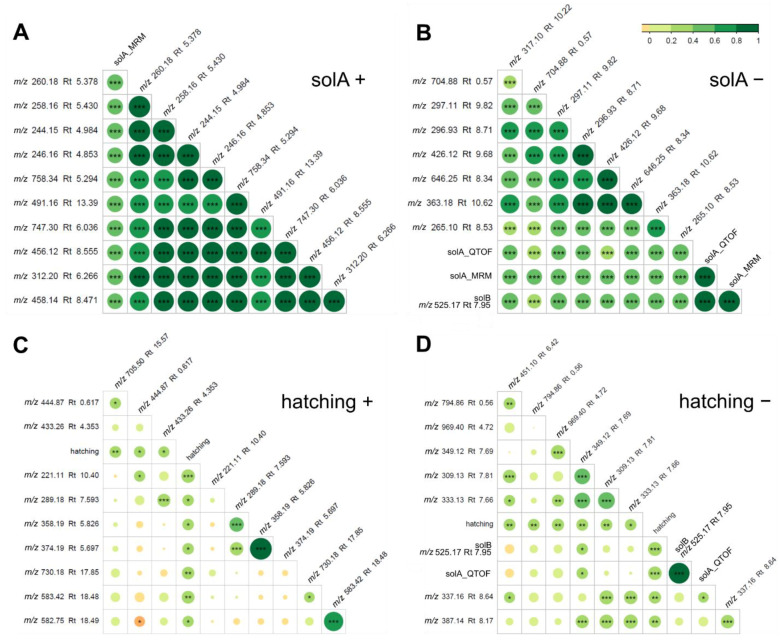

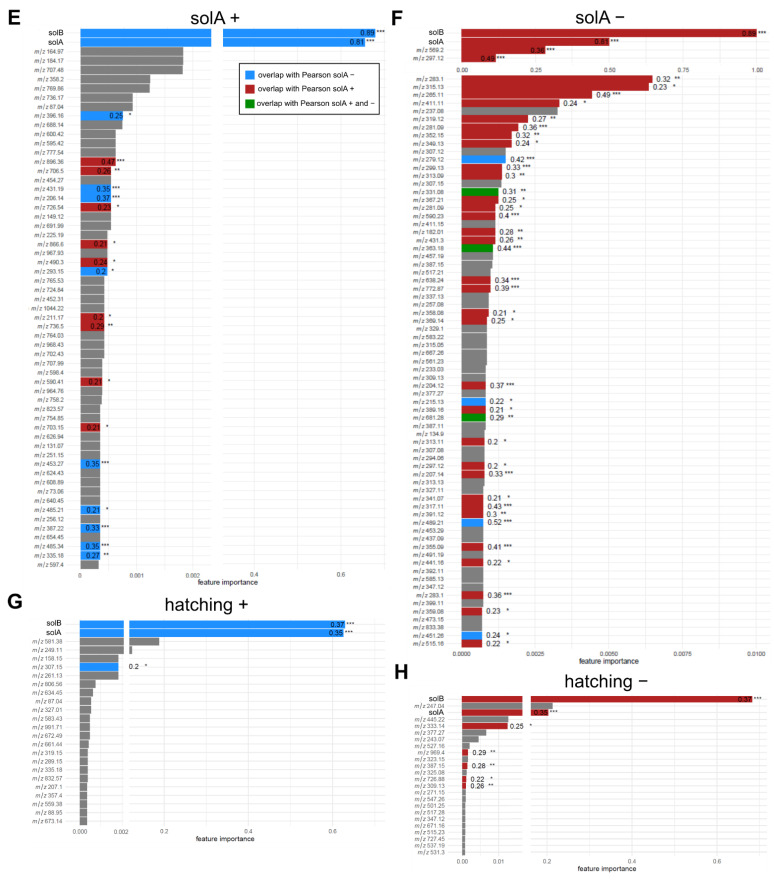
(**A–D**): *m*/*z* values of features in root exudate samples detected by LC-ESI-QTOF-MS analysis linearly correlating (Pearson) with (**A**,**B**) solA and (**C**,**D**) hatching in positive mode (**A**,**C**) and negative mode (**B**,**D**). The features in the (Pearson) correlograms are ordered according to the correlation coefficient using “hclust” method (corrplot package in R). (**E**–**H**): random forest feature selection from LC-ESI-QTOF-MS root exudate data for (**E**) positive and (**F**) negative modes with solA measured by UHPLC-MS/MS (solA_MRM) and positive (**G**) and negative modes (**H**) with hatching. The features that were also detected with Pearson’s correlation analysis, in the same mode (positive or negative), are marked in red. Features that were detected with Pearson’s correlation analysis in the opposite mode are marked in blue. Features that were detected with Pearson’s correlation analysis in the same as well as in the opposite modes are marked in green. Their Pearson’s correlation coefficients are displayed in or next to the bar. The cut-off for the Pearson’s correlation coefficient was 0.1. Only statistically significant (*p* < 0.05) Pearson’s correlations are marked. The degree of significance is marked by stars: *** *p* < 0.001, ** *p* < 0.01, and * *p* < 0.05. Retention times of features in root extracts and root exudates are not comparable, since they were run on a different column, although of the same type.

Next, the relationship between metabolomics features and solA content determined by UPLC-MS/MS analysis was studied using Pearson’s correlation analysis (Figure 2A–D) and RF feature selection (Figure 2E–H), which was statistically validated through 100 permutation tests (*p*-value < 0.05) (Table S2). The RF results in a feature importance score were between 0 and 1 and were calculated from the inverse of the rank. In the Pearson’s correlation test for positive mode, six features, and in negative mode, two features, were highly correlated with solA, with the highest coefficient reaching 0.89 (Table 1). In the RF analysis, 60 and 75 features, in the positive and negative modes, respectively, were selected to contribute significantly (*p*-value < 0.05) to SolA content. Among the features thus selected was also solA that we were able to find back in the metabolomics data (Table 1). This feature correlates, as expected, to a high degree with the solA concentration determined by UPLC-MS/MS analysis (solA_MRM), with a Pearson’s correlation coefficient of 0.81 and RF feature importance values of 0.65 and 0.51 for the positive and negative modes, respectively (Figure 2E,F).

Moreover, a feature [M-H]^−^ 525.1771 (Rt 7.95, Table 1) correlated to an even higher extent with solA (R2 = 0.89, Figure 2B, Table S1) and had an RF feature importance score of 0.67 and 1 in the positive and negative modes, respectively (Figure 2E,F). Intriguingly, the MS2 spectrum of this feature showed strong similarities with the solA MS2 spectrum with several of the same fragments: 481.184, 453.187, and 83.0487 (Figure S2) with the molecular formulas C_27_H_29_O_8_^+^, C_26_H_29_O_7_^+^, and C_5_H_7_O^+^, respectively. The feature has a predicted molecular formula of C_28_H_30_O_10_ and a mass difference of plus 27.9955 Da compared with SolA, which theoretically could correspond to the methylation of one of the alcohols (+14) and double oxidation to a carbonyl (+14) of solA. Since both share the *m*/*z* 83.0487 fragment, the carbonyl may be present on rings A, B, C, D, or E (the allylic position on ring D is a good candidate position), while the methylation may occur on the alcohols of the C or E rings. In conclusion, we expect that this compound will be structurally similar to solA, and we will call this compound solanoeclepin B (solB).

The correlation and predictive value of features for hatching were generally lower, with only one and two moderately correlating features in positive and negative modes and no highly correlating features in Pearson’s correlation tests (Figure 2C,D). The RF feature selection for the correlation with PCN hatching rendered fewer compounds: 24 for both positive and negative modes, of which three and seven were also detected with the Pearson’s correlation test (Figure 2G,H). The highest correlators for Pearson and predictors found in RF are solA and solB (Figure 2C,D,G,H). Interestingly, in the negative mode, the RF feature importance is almost three times higher for solB than for solA (Figure 2H), suggesting that solB predicts hatching better than solA. A further analysis of the results yielded three more interesting features: [M-H]^+^ 581.3835 and [M-H]^+^ 249.1108 had a feature importance > 0.1, and [M-H]^−^ 247.0435 had a feature importance of 0.2 (Figure 2C,D,G,H, Table 1). These features were not significant in the Pearson’s correlation analysis, so they may have a nonlinear correlation with hatching. Features with a feature importance lower than 0.2 were not further considered in this manuscript.

### 2.3. Fractionation of Root Exudates Shows Specific Hatching Activity

**Figure 3:**
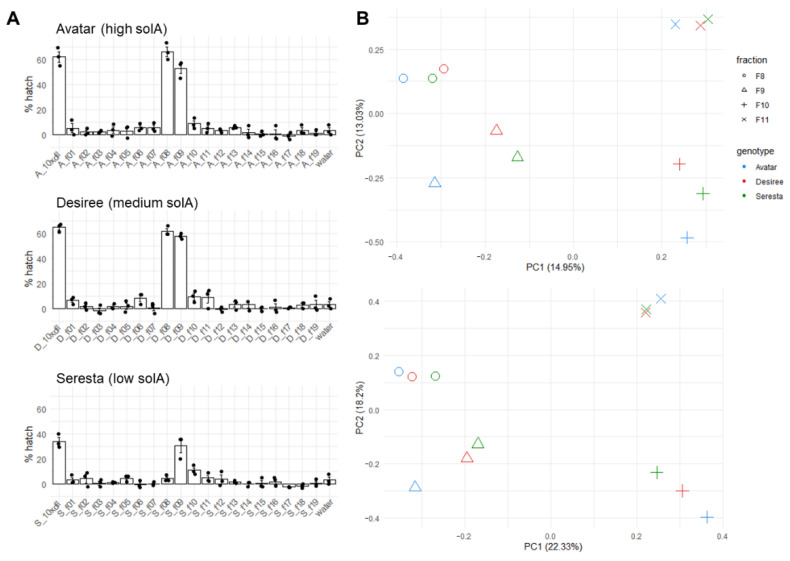
(**A**) Hatching of *G. rostochiensis* eggs under influence of fractions of three root exudates of genotypes Avatar (A), Desiree (D), and Seresta (S). These genotypes had high and low concentrations of solA (Figure 1A). Fractions 8, 9, 10, and 11 were selected for LC-ESI-QTOF-MS analysis. The first column shows the hatching percentage of the 10× diluted unfractionated root exudates. The concentrations of fractions are comparable to the 10× diluted unfractionated root exudates. (**B**) PCA plots of LC-ESI-QTOF-MS data positive (top) and negative (bottom) modes, of four selected fractions associated with high hatch-inducing activity of root exudates of the genotypes Avatar, Desiree, and Seresta. 10 × dil = 10 × diluted.

To confirm the hatching activity of some of the features we identified, three cultivars were selected that induced high (Avatar and Desiree, −21%) and low (Seresta, 10.5%) hatching in PCNs and contained high (Avatar, 95.42 pmol/g FW and Desiree, 81.58 pmol/g FW) and low (Seresta, 9.37 pmol/g FW) levels of solA in their root exudates. These three root exudates were fractionated into 19 fractions using UPLC that were tested for hatching activity (Figure 3A). In Avatar and Desiree, fractions 8 and 9 induced hatching comparable to the unfractionated root exudate (approx. 60%). Furthermore, fractions 10 (both genotypes) and fractions 11 (only Desiree) also induced significant hatching. For Seresta, fraction 9 induced similar hatching to the unfractionated root exudate, while fraction 10 also induced some hatching (Figure 3A).

### 2.4. Metabolomics of Fractions with High Hatching Activity Confirm Discovery of a New Hatching Factor

**Figure 4:**
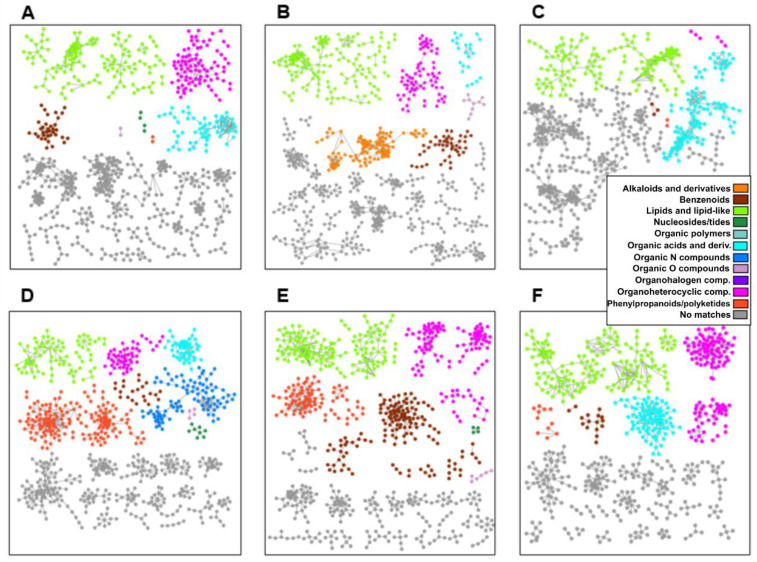
Classical GNPS molecular networks of Avatar (**A**,**D**), Desiree (**B**,**E**), and Seresta (**C**,**F**) root exudate LC-ESI-QTOF-MS data, analyzed in positive (**A**–**C**) and negative modes (**D**–**F**). Networks were constructed using default settings. Nonconnected nodes and gray networks consisting of 4 or fewer nodes were deleted to improve visibility.

To assess if we could detect the presence of some of the putative hatching factor candidates identified above, in the hatch-inducing fractions, fractions 8, 9, 10, and 11 were analyzed using LC-ESI-QTOF-MS. The PCA analysis shows that in both positive and negative modes, the same fractions of the different genotypes cluster together, although the combined cumulative contribution rate of the first two components is not high (Figure 3B). This means that the fraction explains the variation in the metabolite profiles only partly, and other factors, among which there is possibly the genotype, explain the rest of the variation. Using the GNPS tool, classical molecular networks were constructed from the LC-ESI-QTOF-MS data of these three cultivars (Figure 4). This showed that the three genotypes are chemically different, especially in the amount of organic acids and derivatives. Avatar exudate contains the most phenylpropanoids and polyketides, followed by Desiree. Desiree exudate contains many more benzenoids and alkaloids than the other two cultivars.

**Figure 5:**
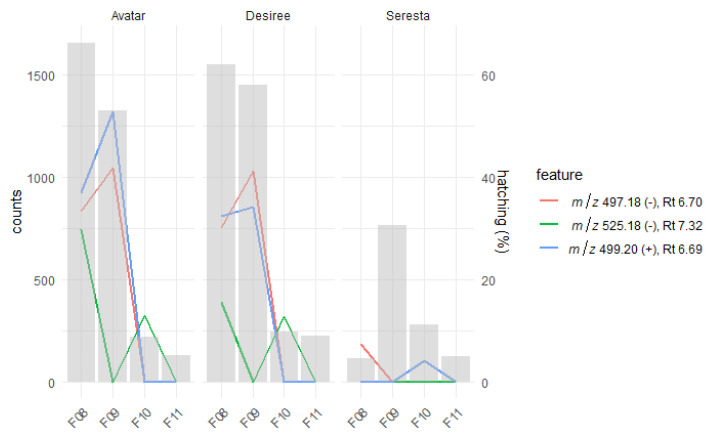
Count levels of individual features per fraction per genotype that were selected through Pearson’s correlation, RF feature selection, and their presence in the active fractions of root exudates. Line plots show the count levels of individual features, whereas the gray bars show the % hatching of PCN of the respective fraction induced. For solA, a retention time shift was observed between this experiment and the previous, whole root exudate analyses (Figure 2), 6.70 to 7.66 min.

Several *m*/*z* values that were selected for their hatching activity by Pearson’s correlation and RF were detected in these fractions as well (Table 1). Among these are solA ([M-H]^−^ 497.18199) and solB ([M-H]^−^ 525.17611), which highly correlated with both hatching and solA presence in the complete root exudates (Figure 2). Comparing the relative abundance of these compounds in the fractions with the PCN hatching shows that solA, which is detected in both positive and negative modes, is mostly present in fractions 8 and 9 (Figure 5). In Avatar and Desiree, these fractions induced the most hatching. In Seresta, only a trace amount of solA was detected (the metabolomics analysis is less sensitive for SolA than for the MRM-LC-MS/MS analysis). Since solA concentrations are the highest in fraction 9, but the highest hatching in Avatar and Desiree is induced by fraction 8, there likely is an additional HF present in the latter fraction. Indeed, [M-H]^−^ 525.17611 is present in fraction 8 in Avatar and Desiree (Figure 5). Moreover, it seems the hatching activity in fraction 10 of Avatar and Desiree is caused by solB, as no solA was found. We thus considered that solB has hatch-inducing activity. The presence of a trace amount of solB in both fraction 8 and fraction 10 suggests these are isomers, which are separated by UPLC but not separated by the LC-ESI-QTOF-MS. The relative abundance of this compound in the twenty cultivars that were metabolically analyzed is highly variable (Figure S3): the highest producer, cultivar Avatar, contains about ten times more of this compound than the root exudate of the lowest producer, Ivory Russet. Just as there is natural variation in solA concentration in different cultivars, so too does solB display natural variation among the tested cultivars (Figure 5).

Apart from solA and solB, there were other compounds present in fractions 8, 9, 10, and 11 that highly correlate with hatching within the scope of these fractions (Figure S4). Interestingly, the compound that had the highest correlation with hatching as well as solA content in whole root exudates, solB, is not within the top 10 in the fraction correlation. The highest correlating compound with hatching was solA, followed by the compounds [M-H]^−^ 297.13419 and [M-H]^−^ 293.10245. These compounds were not found to correlate with hatching in the whole root exudate dataset. However, compounds that scored high in this analysis might have done so because they have similar chemical properties to solA and therefore ended up in these fractions and might have nothing to do with hatching. This analysis would benefit from including all fractions and their LC-ESI-QTOF-MS analysis, but this was not attempted in this study.

### 2.5. Root Extracts Do Not Contain SolA, but Some Metabolites That Correlate with Hatching May Be Its Precursors

Because the hatching factors are produced in and, subsequently, exuded from the roots, we also performed metabolomics on root extracts, hoping to identify additional features associated with solA and hatching. Roots were ground, extracted, and subsequently analyzed by LC-ESI-QTOF-MS. A Principal Component Analysis (PCA) showed reasonable separation of the cultivars for the negative mode and, to a lesser extent, for the positive mode (Figure S5). PC1 and PC2 together explained 16.84% and 25.23% of the variation in the metabolic features measured in negative and positive modes, respectively.

**Figure 6:**
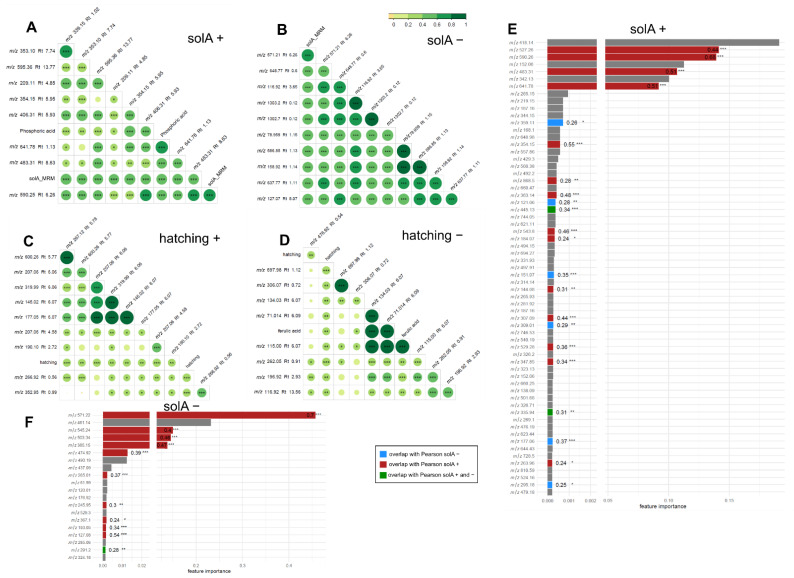
*m*/*z* values and retention times (Rt) of compounds in root extract samples detected by LC-ESI-QTOF-MS analysis that correlate with solA measured by UHPLC-multiple reaction monitoring-MS/MS (solA_MRM) (**A**,**C**,**D**) content and/or hatching (**B**), detected with Pearson’s correlation test (**A**–**D**) and Random Forest feature selection methods (**E**,**F**). (**A**) correlograms showing linear Pearson’s correlations of metabolites with solA_MRM in positive (**A**) and negative (**B**) mode. (**C**,**D**) correlograms showing linear Pearson’s correlations of metabolites with hatching in positive (**C**) and negative (**D**) mode. Order of the features in all correlograms is reordered according to the correlation coefficient using “hclust” method (corrplot package in R). (**E**) random forest feature selection for positive mode with solA_MRM. (**F**) random forest feature selection for negative mode with solA_MRM. The features that were as well detected with Pearson’s correlation analysis in the same mode (positive or negative) are marked in red. Features that were detected with Pearson’s correlation analysis in the opposite mode are marked in blue. Features that were detected with Pearson’s correlation analysis in the same as well as the opposite mode are marked in green. Their Pearson’s correlation coefficient is displayed in or next to the bar. The threshold for the Pearson’s correlation coefficient was 0.1. Only statistically significant (*p* < 0.05) Pearson’s correlations are marked. The degree of significance is marked by stars: *p* < 0.001, ***; *p* < 0.01, **, *p* < 0.05, *.

SolA was not detected in root extracts or when using MRM-LC-MS/MS, but Pearson correlation and feature selection by random forest (RF) machine learning yielded several features that significantly correlated with solA content in the root exudate and PCN hatching. The top 10 Pearson correlating compounds are displayed in correlograms (Figure 6A–D. Pearson correlation with solA concentration resulted in 7 and 12 highly correlated compounds (coefficient > 0.5) in positive and negative modes, respectively (Table S1). The two features with the highest coefficients (0.70 and 0.68) are [M-H]^−^ 571.2182 and [M-H]^+^ 590.2600, respectively (Figure 6A,B, Table 1 and Table S1). The correlation coefficient of root extract features with hatching was lower, less than 0.5. A moderate degree of correlation (coefficients between 0.3 and 0.5) was found for 14 and 8 compounds for the positive and negative modes, respectively. The two features with the highest correlation with hatching were [M-H]^−^ 262.0568 (coefficient 0.36) and [M-H]^+^ 266.9259 (coefficient 0.37) (Table 1 and Table S1). These two compounds had very short retention times, which indicates they are polar. The top correlating compounds with solA and hatching did not overlap.

Whereas Pearson’s correlation only detects linear correlations, feature selection by RF machine learning can also detect other types of relationships. The RF results on predictive features for hatching could not be validated by permutation tests (Table S2). Therefore, we only used RF feature selection for solA content, which could be validated by permutation tests (Table S2, Figure 6E,F). In positive mode, 63 features, and in negative mode, 20 features were selected. Out of these, 27 and 14, respectively, were also found in the Pearson’s correlation test (Figure 6). The two compounds with the highest Pearson’s correlation coefficient ([M-H]^−^ 571.2182 and [M-H]^+^ 590.2600) were confirmed with RF (Table 1, Figure 6E,F). Upon further inspection, the compound [M-H]^−^ 571.2182 has an exact mass of 572.2254 and is likely C_30_H_35_O_11_ (Table 1). It could possibly be a solA precursor (Figure S6), such as a cycloartenol-derived abietospiran-type precursor with a C30 backbone, as suggested in Sun et al. (2019) [14]. However, due to the low amount that was detected, we cannot show a reliable MS2 spectrum.

## 3. Discussion

In the present study, we used a new approach to detect putative HFs and HSs for PCNs produced by commercial potato cultivars. This approach involved the use of a large number of potato cultivars and metabolomics in combination with machine learning, RF feature selection, and Pearson’s correlation analysis to uncover correlations between metabolic features and hatching. The involvement of several highly correlating features in hatch stimulation was subsequently confirmed by the fractionation of representative root exudates. With this, we contribute to the elucidation of the activity of specific compounds in the hatch-inducing cocktail exuded by plant roots and thereby to possible solutions for this important agricultural problem, for example, by screening cultivars for the level of HF production and selecting low producers. First, it was shown that solA content and hatching percentage of root exudates are logarithmically correlated, but this correlation is weak, which suggests that more HFs and HSs, and possibly HIs, are influencing hatching. In the next experiment, root exudates were analyzed through metabolomics on LC-ESI-QTOF-MS, and features were computationally linked to solA content and hatching by Pearson’s linear correlation test and RF (Figure 2). A compound of molecular weight 526.17 correlated with solA and hatching to a higher extent than solA itself. This exceptionally high correlation (correlation coefficient of 0.89 and RF feature importance of 1, which is the highest possible) suggests that this compound is structurally related to solA. A comparison of the MS2 spectra of this compound, coined solB, with solA revealed three identical fragments lending support to the postulated structural relatedness (Figure S2). Lastly, we fractionated three selected root exudates that varied in solA concentration and hatch-inducing potency. Even though the hatch-inducing activity between cultivars varied greatly, in PCA, the same fractions of different cultivars clustered together (Figure 3B). Hence, only a small number of compounds in root exudates influence hatching, and their hatch-inducing potency is not a major discriminating factor. SolA and solB were both found in fractions inducing high PCN hatching rates (Figure 5). Next, the root extracts from twenty cultivars were analyzed by untargeted metabolomics to find solA/solB precursors and/or other HFs. Through Pearson’s correlation test and RF, we could correlate the root metabolite features with solA content of the root exudate, some of which showed a high correlation (Figure 6). The root extracts did not contain solA, but they might have contained precursors of solA, of which the concentration could correlate with solA content in the exudate. Indeed, especially for a feature of 572.2 Da, a strong correlation with solA content was detected in both data analysis methods. The predicted structural formula of this feature has A, B, and C rings that are identical to those of solA (Figure S6). The correlation of root extract features with hatching was low, and the RF models were not significant.

On a side note, in this study it was observed that hatching with unpurified but diluted root exudates results in almost three times more (60% vs. 21% for Avatar and Desiree and 35% vs. 10.5% for Seresta) hatching than the partially purified root exudates (Figure 1B and Figure 3A). This was shown before for Sephadex G-10-fractionated root exudates [15]. This is probably because HFs work according to an optimum, and concentrations above this optimum do not stimulate or even inhibit hatching [16]. Furthermore, by dilution of root exudates, HIs are diluted as well, which reduces their inhibitory effect. This effect might play a role as well for high hatch-inducing fractions (Figure 3A), which contain mainly HFs and HSs, whereas the HIs might end up in a different fraction based on their chemical properties and hence do not mitigate the hatch induction of the fraction.

Other metabolic features that showed to be interesting in our data analyses (Table 1) are hard to identify, partially because of low mass intensities that resulted in poor quality MS2 spectra. So far, we can conclude that they do not overlap with previously purified but unidentified hatching factors, such as a 530.5 Da compound identified by Devine and Jones [17] or the 437 Da compound identified by Atkinson et al. [18]. The molecular weights we found in the current study also do not match with glycoalkaloids identified in potato (for example, α-chanonine or α-solanine), which all have molecular weights (MWs) of over 800 Da, or their aglycones (solanidine, MW 397.6 and solasodine, MW 413.6).

The other eclepin group, the glycinoeclepins produced by kidney bean, displays more structural variation, among which glyB and glyC were reported to be 1000-fold less active than glyA in inducing the hatching of SCN [10,11]. A plant genotype that produces a high amount of glyB and glyC but a low amount of glyA will likely induce little SCN hatching. This could imply that kidney bean has evolved new, slightly different structures to avoid their chemical signaling being abused by SCN. We thus anticipated that more solanoeclepins also exist with different hatching activity than solA. The diversification of eclepins, therefore, could be caused by a race between plant and parasitic nematode. Since the eclepins have a detrimental effect on the plant (the cyst nematode hatch induction) but are indispensable at the same time because of a hitherto unknown beneficial effect, the plant’s eclepin biosynthesis diversifies, leading to the biosynthesis of new eclepins that still have the beneficial effect but induce no or lower cyst nematode hatching, thus conferring resistance to cyst nematodes. The same phenomenon was also observed in the plant–parasitic plant interaction. Sorghum genotypes with a high ratio of the strigolactones orobanchol/5-deoxystrigol induce much less germination of the parasitic plant Striga than genotypes with the reverse and are therefore to a large extent resistant [19]. It was found that the mutation of the strigolactone biosynthetic gene LOW GERMINATION STIMULANT 1 resulted in a change in strigolactone composition in sorghum root exudates. This example suggests that parasites abuse plant-produced chemicals and that plants evolve new chemical structures to avoid this, likely by adapting biosynthetic enzymes. In another example, *Barbarea vulgaris* has diverged to two chemotypes (G- and P-types). The G-type of *B. vulgaris* is more resistant to insects than the P-type because it produces different saponin structures [20]. In this paper, we demonstrated the existence of different structures of solanoeclepins in potato root exudates and observed natural variations of both solA and solB in potato cultivars. This is the first step toward understanding the complexity of PCN hatching and the coevolution of the potato host and its parasitic PCN species. This offers the basis for further structure elucidation of solB, as well as a comparison of hatching activities between different solanoeclepins.

In the present study, the influence of microorganisms on the production of hatching factors and stimulants was not monitored. However, microorganisms have been reported to have a considerable effect on the hatch-inducing cocktail. For example, mycorrhization increases the production and variety of hatching factors in potato roots [21] and aseptically grown potato plants lack hatching factors that are present in the root exudate of conventionally grown potato plants [22]. Indeed, it is noteworthy that solA could not be detected in root extract, only in exudate, which suggests that it is exuded as a precursor and metabolized into solA by microorganisms. Therefore, follow-up experiments should take this factor into account by, for example, analyzing root exudates from aseptically grown plants. For the current study, it can be assumed that all compounds produced by microorganisms that are present in bulk soil, so independent of the plant, were filtered from the dataset (through the use of an empty pot control). Hence, all features discussed in this manuscript depend on the presence of potato, either directly (biosynthesis in the plant) or indirectly (biosynthesis by potato root-associated microorganisms).

The PCN is a pest that causes severe damage and yield losses in potato. By identifying the compounds that induce its hatching, it becomes possible to select for potato cultivars that produce low concentrations of hatching factors, thereby preventing hatching and thus infestation of the roots by PCNs. For example, from the cultivars that were analyzed in our study, Ivory Russet and Seresta produced the lowest concentrations of solA and solB (Figure 1 and Figure S3), which would make them good candidates for breeding low HF producers. Furthermore, other plant species that produce these hatching factors, but are not a suitable host for PCNs, can then be identified. These species can be used as trap crops, inducing suicide hatching in PCNs. This was observed before in the wild tomato relative, *Solanum sisymbriifolium*, one of the best-known PCN trap crops that we recently showed to produce solanoeclepin A, but is not a host for PCN [9,23].

## 4. Materials and Methods

### 4.1. Chemicals

A standard of solanoeclepin A was kindly provided by Prof. Keiji Tanino (Hokkaido University, Japan). KOH and NH_4_OH for sample extraction and purification were obtained from Merck KGaA (Darmstadt, Germany). Methanol, acetonitrile, deionized water, and formic acid for LC-ESI-QTOF-MS and UPLC-MS/MS analyses were all hypergrade for LC-MS (Biosolve BV, Valkenswaard, The Netherlands). Milli-Q water was prepared using a water purification system Milli-Q^®^ (Merck Millipore, Burlington, MA, USA).

### 4.2. Plant and Nematode Materials and Growth Conditions

Tubers of *S. tuberosum* were obtained from three different potato breeding companies: Avebe (Veendam, The Netherlands), HZPC Holland B.V. (Joure, The Netherlands), and Meijer Potato (Rilland, The Netherlands). Cysts of *G. rostochiensis* Mierenbos pathotype Ro1 were obtained from Aska Goverse, Laboratory of Nematology, Wageningen University. Cysts were reared in the greenhouse, and dormancy was broken by storage in −80 °C for several months, after which they were stored at 4 °C until use.

Plants were grown in the greenhouse in pots (8 cm bottom diameter, 12 cm top diameter, and 9 cm height) with standard greenhouse compost no. 3, using one tuber per pot. Root exudates from the complete root system were collected from 5 replicates for each cultivar by pouring distilled water on the soil in the pot and collecting 200 mL of flow-through per pot. The root exudates were stored at 4 °C until further processing. Roots were collected from the soil; all soil particles were carefully removed through washing, and the roots were dried with paper tissues and subsequently stored at −80 °C.

### 4.3. Sample Extraction and Purification

Root exudates were filtered over filter paper to remove soil particles. Root exudates for solA measurement were purified by solid phase extraction (SPE) using vacuum through Oasis^®^ MAX cartridges (3 cc/60 mg, Waters, Milford, MA, USA) according to the protocol from Guerrieri et al. [9]. For metabolomics analysis, C18 cartridges (6 cc/500 mg, Waters, Milford, MA, USA) were conditioned and equilibrated as per the manufacturer’s instructions. Of each sample, 25 mL was loaded on the cartridge, after which the cartridge was washed with 3 mL MQ. Elution was achieved with 3 mL methanol. Samples were dried under vacuum, redissolved in 20% methanol, and filtered using 750 uL 0.22 μm Nonsterile Micro-Centrifugal Filters (Fisher Scientific, Landsmeer, The Netherlands) before LC-ESI-QTOF-MS analysis.

Roots were ground to a fine powder using liquid N_2_, and subsequently 100 mg of this powder was extracted with 80% methanol according to the protocol from De Vos et al. [24]. Extracts were filtered using 750 μL 0.22 μm Nonsterile Micro-Centrifugal Filters (Fisher Scientific, Landsmeer, Netherlands) before LC-ESI-QTOF-MS analysis.

### 4.4. UPLC-Multiple Reaction Monitoring-MS/MS Analysis

SolA analysis was executed according to Guerrieri et al. [9]. In short, 5 µL of purified root exudate was injected onto the reversed-phase UPLC column (Acquity UPLC^®^ Ethylene Bridged Hybrid (BEH) C18 column, 2.1 × 100 mm, 1.7 μm particle size, Waters), and the eluent was introduced into the ESI ion source of the mass spectrometer. SolA was analyzed in positive mode as [M+H]^+^, using transitions 499 > 83, 499 > 315, 499 > 399 and 499 > 453. The instrument MS data acquisition and processing were carried out by MassLynxTM software, version 4.2 (Waters, Milford, MA, USA). The resulting solA concentrations in pmol/g FW refers to the weight of the roots that exuded this amount of solA.

### 4.5. LC-ESI-QTOF-MS Analysis

Untargeted metabolomics was executed according to Guerrieri et al. [9]. Briefly, root exudates and extracts and UPLC fractions were analyzed using a QTOF-MS equipped with a dual-stage trapped ion mobility separation cell (timsTOF pro Bruker Daltonics Inc., Billerica, MA, USA). Sample injection (20 μL) and LC separation were performed on an Ultimate RS UPLC system (Thermo Scientific, Germeringen, Germany) with an Acquity UPLC CSH C18 130 Å, 1.7 µm, 2.1 mm × 100 mm protected by a VanGuard 2.1 mm × 5 mm of the same material. A gradient from 1% acetonitrile to 99% acetonitrile in 18 min was applied. Eluting compounds were sprayed in positive and negative ion modes by an Apollo II ion funnel ESI source (Bruker Daltonics Inc.). The resulting data were analyzed using DataAnalysis ver. 4.3 (Bruker Daltonics) and MetaboScape^®^ 5.0 (Bruker Daltonics).

### 4.6. Fractionation of Root Exudates

For UPLC fractionation of root exudates, 50 mL of the exudate was lyophilized, and salts were precipitated by dissolving the dried exudate in 3 mL of methanol. The samples were centrifuged at 4000 rpm for 3 min, and the supernatant was transferred to another vial after which it was dried by vacuum evaporation. Subsequently, the residue was reconstituted in 150 µL 25% acetonitrile and injected onto the same Waters UPLC equipped with the same column, as described above. Separation of the samples was achieved using the same gradient as used for solA quantification. The eluent was fractionated using an analytical fraction collector (WFM-A) for UPLC systems and the operating software Empower v.3.6.0 (Waters, Milford, MA, USA). Each sample was injected 8 times, and 19 pooled 30 sec fractions were collected between 0.5 and 10 min. Of each fraction, 12 µL was lyophilized and then diluted 33-fold to a volume of 0.4 mL in 2% ethanol and then used to test hatching activity. This resulted in the fractions being 33-fold more concentrated than the crude root exudate, which is similar to the concentration used for UPLC-MS/MS analysis, obtained through SPE sample processing. The remaining fraction sample was freeze dried once again and dissolved in 150 µL 25% ACN for untargeted metabolomics using LC-ESI-QTOF-MS.

### 4.7. Nematode Hatching Assay

Hatching assays were carried out as described in Guerrieri et al. [9]. In short, cysts were hydrated for 7 days, and, subsequently, eggs were released from the cyst by manually opening each cyst separately. A total of 100 µL of egg suspension, containing around 100 eggs, was distributed to the wells of a glass-coated 96-well plate. Subsequently, 100 µL of test solution (dried root exudate that was filtered and partially purified by SPE dissolved in 2% EtOH in tap water, dried fraction of root exudate dissolved in 2% EtOH in tap water, and solA dissolved in 2% EtOH in tap water) was added to each well. Eggs and juveniles were counted at t = 0 and t = 14 days. Hatching percentage was calculated according to the formula:

(J_t14_ − J_t0_)/E_t0_ × 100

where J_t11_ is the number of hatched J2 after 14 days of treatment; J_t0_ is the number of hatched J2 at the start of the assay, and E_t0_ is the number of eggs at the start of the assay.

### 4.8. Data Analysis

The resulting feature table was filtered by removing features with intensities lower than or equal to the empty pot control (both average of five replicates), occurring in less than three replicates for any cultivar. Furthermore, peak areas below 200 were considered to be absent. For Principal Component Analysis (PCA), feature tables were transformed using the variance stabilizing normalization (vsn) method and scaled using autoscaling method. This data preprocessing was performed with the packages vsn (v3.58.0) [25] and mdatools (v0.12.0) [26] using R (v4.0.2) in Rstudio (v1.3.1093). Furthermore, the package ggplot2 (v3.3.5) was used to plot the trendline of Figure 1 [27]. Pearson’s correlation plots, which show the correlation between solA content and metabolite features and the hatching percentages and metabolite features, were assembled using the package corrplot (v0.90) [28]. Only the top 10 correlating features are displayed.

The log2 transformed metabolomic data were used to build a multivariate model using a random forest-based MUVR (v0.0.975) in R [29]. Variable selection and validation in a multivariate model were conducted using an algorithm that simultaneously identified minimal, optimal, and all relevant variables for regression analysis. The model was built using the following parameters: nRep = 14, nOuter = 8, and varRatio = 0.85. The model was evaluated with the fitness estimator Q2, which was used for regression analysis, whereas the number of misclassifications was used for classification and multilevel analysis.

Permutations were obtained by 100 and 150 (exudates and root extract, respectively) times repeated random sampling of the original metabolomic matrix. To assess modeling performance, permutation *p*-values were thus calculated with the population of fitness metrics.

Molecular networks were calculated using the online platform Global Natural Products Social Molecular Networking (GNPS) using the Molecular Networking option [30]. Raw mzXML files of the LC-ESI-QTOF-MS data of the fractions 8, 9, 10, and 11 of cultivars Avatar, Desiree, and Seresta were uploaded to their server and networks, separated by cultivar and were constructed using Small Data Preset and further default settings. The resulting network was further modified using MolNetEnhancer (default settings) [31], downloaded, and opened in Cytoscape (v3.9.0) [32] for rendering.

Detailed scripts can be found in R markdown files in the Supplemental materials.

## 5. Conclusions

In this study, we demonstrated that machine learning combined with metabolomics and hatching assays is a useful method to detect new HFs and/or HSs. Furthermore, we showed that potato cultivars produce a hitherto unknown compound, solB, which highly correlates with the hatching rate of PCNs and the presence of solA. This work establishes the foundation for a further selection of cultivars with low HF production, inducing low hatching of PCNs, thereby contributing to more sustainable agriculture.

## Figures and Tables

**Table 1:** Metabolites detected in root extract, exudates, and fractions of exudates that are of particular interest because of high correlation (RF and/or Pearson’s) with solA content of root exudates or PCN hatching. Molecular *m*/*z* is the theoretical mass to charge ratio, whereas measured *m*/*z* is the detected mass to charge ratio under positive or negative ionization modes. SolA and solB were both detected under both positive and negative ionization modes. Δ *m/z* (ppm) is calculated by the formula (theoretical *m*/*z* value − measured *m*/*z* value)/theoretical *m*/*z* value × 10^6^.

Molecular *m/z*	Rt	Measured *m/z*	Calculated Mass	Δ *m/z* (ppm)	Ion	Predicted Formula	Detected in	Putative Name	Correlation
**248.0508**	9.86	247.0435	247.0448	5.4	[M-H]^−^	C_9_H_12_O_8_	Exudate		hatch
**248.11**	6.38	249.1108	249.1121	5.4	[M-H]^+^	C_14_H_17_O_4_	Exudate	prenyl caffeate	hatch
**263.0641**	0.91	262.0568	262.0557	−4.0	[M-H]^−^	C_9_H_13_NO_8_	Extract	ascorbalamic acid	hatch
**265.9186**	0.56	266.9259			[M-H]^+^		Extract		hatch
**498.1887**	7.66	497.1814	497.18061	−1.6	[M-H]^−^	C_27_H_30_O_9_	Exudate, fractions	solA	solA, hatch
**498.1887**	7.66	499.1944	499.1963	3.7	[M-H]^+^	C_27_H_30_O_9_	Exudate, fractions	solA	solA, hatch
**526.1843**	7.95	525.1771	525.1755	−3.0	[M-H]^−^	C_28_H_30_O_10_	Exudate, fractions	solB	solA, hatch
**526.1843**	7.94	527.1893	527.1912	3.6	[M-H]^+^	C_28_H_30_O_10_	Exudate, fractions	solB	solA, hatch
**572.2254**	6.26	571.2182	571.2174	−1.4	[M-H]^−^	C_30_H_36_O_11_	Extract	solA precursor?	solA
**580.38**	13.95	581.3835	581.3837	0.3	[M-H]^+^	C_36_H_52_O_6_	Exudate	hugonone A	hatch
**589.2527**	6.26	590.2600	590.2596	−0.7	[M-H]^+^	C_30_H_39_NO_11_	Extract	platencin A13	solA

## Data Availability

Data and R scripts used in this study are available at https://zenodo.org/badge/latestdoi/320565416, accessed on 2 May 2022.

## Supplementary Materials

The following supporting information can be downloaded at: https://www.mdpi.com/article/10.3390/metabo12060551/s1, Figure S1: PCA plots of positive (A) and negative (B) modes of LC-ESI-QTOF-MS data from root exudate samples of selected genotypes; Figure S2: MS2 spectra of positive mode solA and positive and negative modes of tentative solB including the putative molecular structure; Figure S3: Mass intensity (corrected for root weight) of compound with *m*/*z* 525.18 (-) in all LC-ESI-QTOF-MS-analyzed samples; Figure S4: The top 10 Pearson’s correlations between metabolites in positive and negative modes detected by LC-ESI-QTOF-MS analysis of fractions 8, 9, 10, and 11 of Avatar, Desiree, and Seresta cultivars and hatching of PCNs; Figure S5: PCA plots of positive and negative modes of LC-ESI-QTOF-MS data from root extract samples of selected genotypes; Figure S6: Putative structure of compound detected in root extract that highly correlates with solA as well as hatching with m/z 571.22 (-). Table S1: Significantly (*p* < 0.05) correlating (Pearson) features with solA and hatching in both positive- and negative-mode LC-ESI-QTOF-MS data; Table S2: *p*-values of random forest permutations.
