# Supplementary material for: A Combination of Metabolomics and Machine Learning Results in the Identification of a New Cyst Nematode Hatching Factor
Source: Metabolites. 2022 Jun 16;12(6):551. doi: 10.3390/metabo12060551 (PMC9229034; doi:10.3390/metabo12060551)
Supplement: Supplementary file 1 [file metabolites-12-00551-s001.zip › metabolites-1733342-supplementary/Suppl.materials.pdf]

Supplemental materials

Table S1 (excel xlsx document). Significantly (p < 0.05) correlating (Pearson) features with solA and hatching in both positive and negative mode LC-ESI-QTOF-MS data. The cut-off was a correlation coefficient of 0.10. Features with a high degree of correlation (Pearson's correlation coefficient > 0.5) are coloured in red. Features with a moderate degree of correlation (Pearson's correlation coefficient 0.3 – 0.5) are coloured in orange. Features with a low degree of correlation (Pearson's correlation coefficient < 0.3) are coloured in yellow.

Table S2: p values of Random Forest permutations

dataset	p value	# permutations
exudate (+) hatching	0.042422482	100
exudate (+) solA	0.042422482	100
exudate (-) hatching	0.045679047	100
exudate (-) solA	0.042422482	100
extract (+) hatching	0.056267724	150
extract (+) solA	0.042422482	150
extract (-) hatching	0.150932136	150
extract (-) solA	0.042422482	150

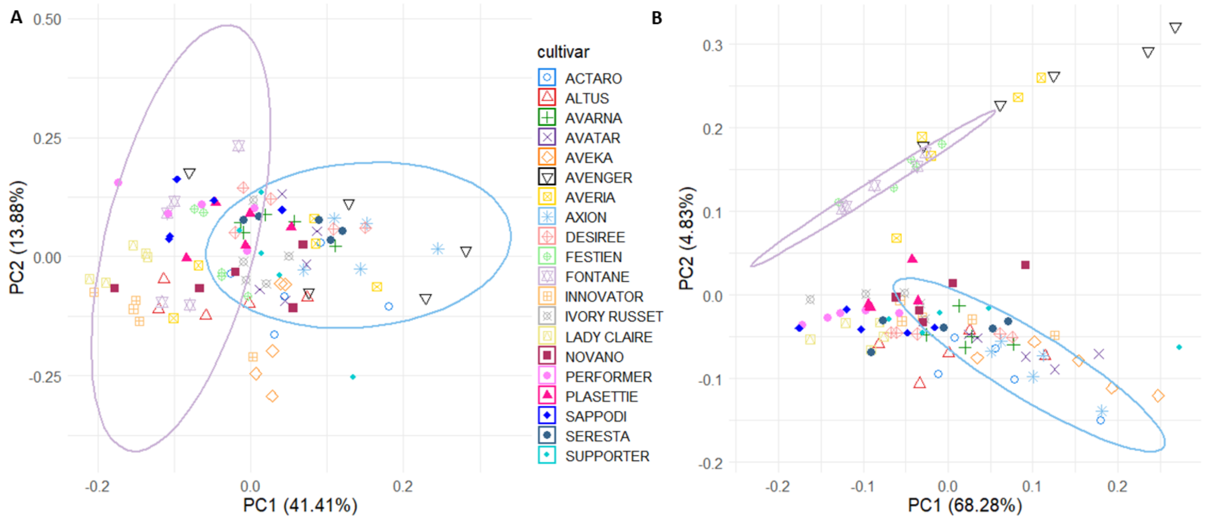

Figure S1: PCA plots of positive (A) and negative (B) mode of LC-ESI-QTOF-MS data from root exudate samples of selected genotypes (Figure 1A). As an example, it is shown that the root exudates of cultivars Axion and Fontane vary metabolically.

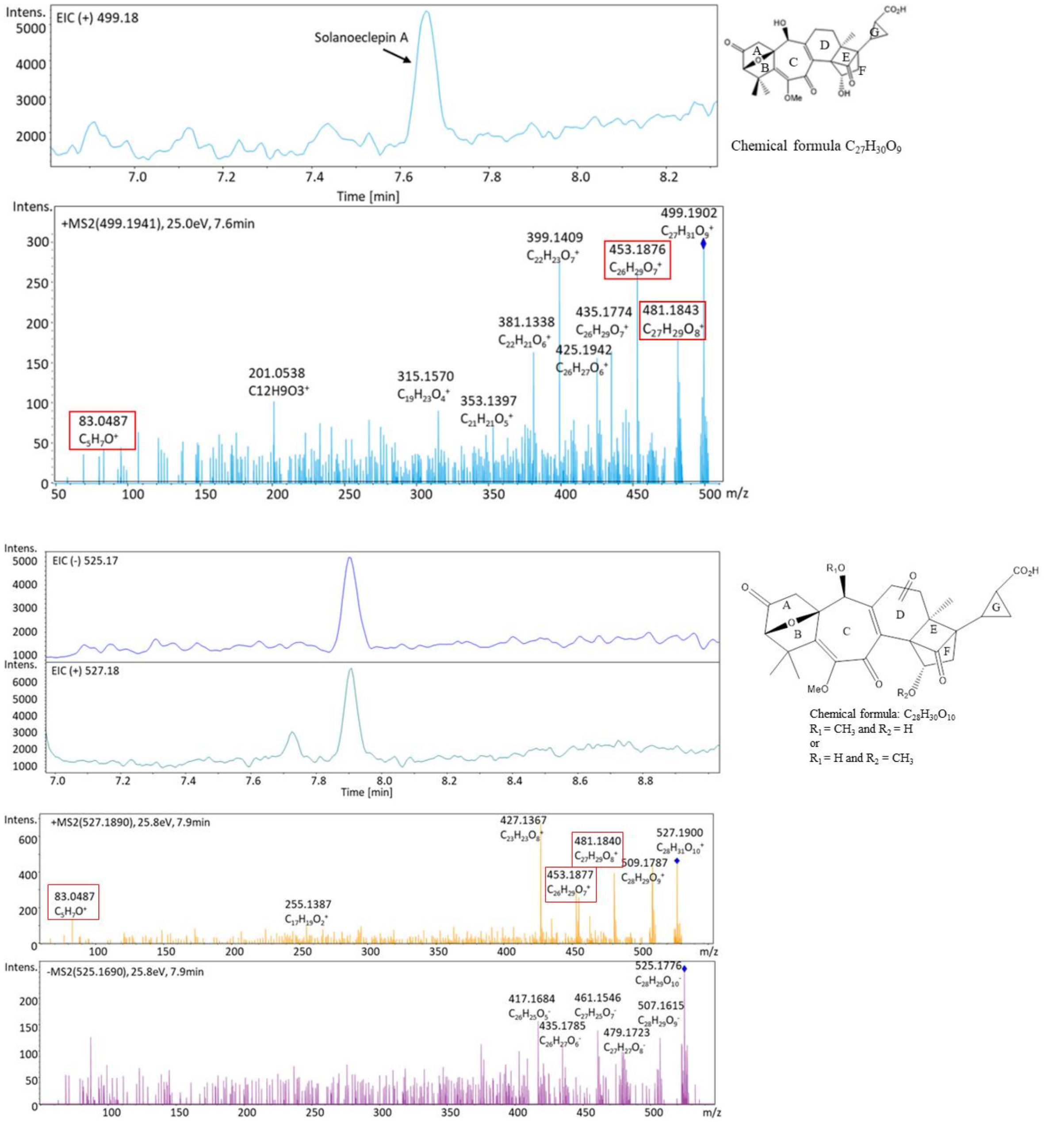

Figure S2: MS2 spectra of positive mode solA (top) and positive and negative mode of tentative solB (bottom) including the putative molecular structure. These were extracted from whole root exudate LC-ESI-QTOF-MS data. Overlapping fragments between these two metabolites are marked with a red box.

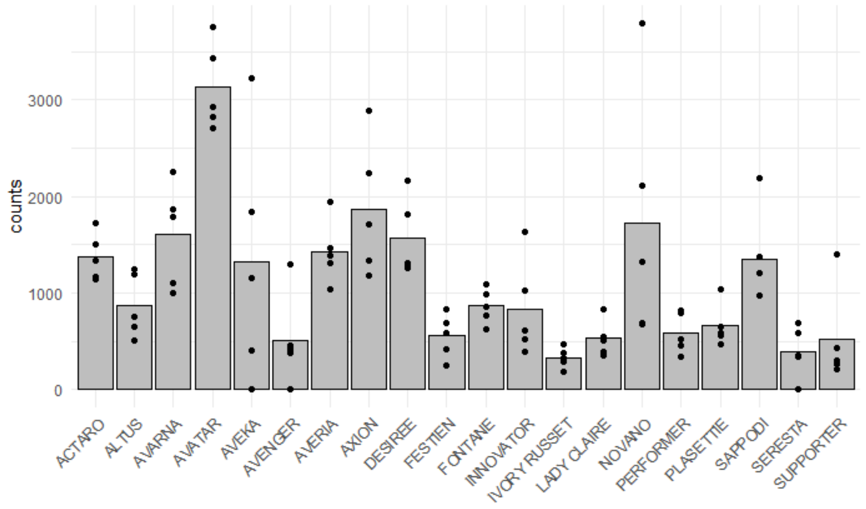

Figure S3: mass intensity (corrected for root weight) of compound with m/z 525.18 (-) in all LC-ESI-QTOF-MS analysed samples

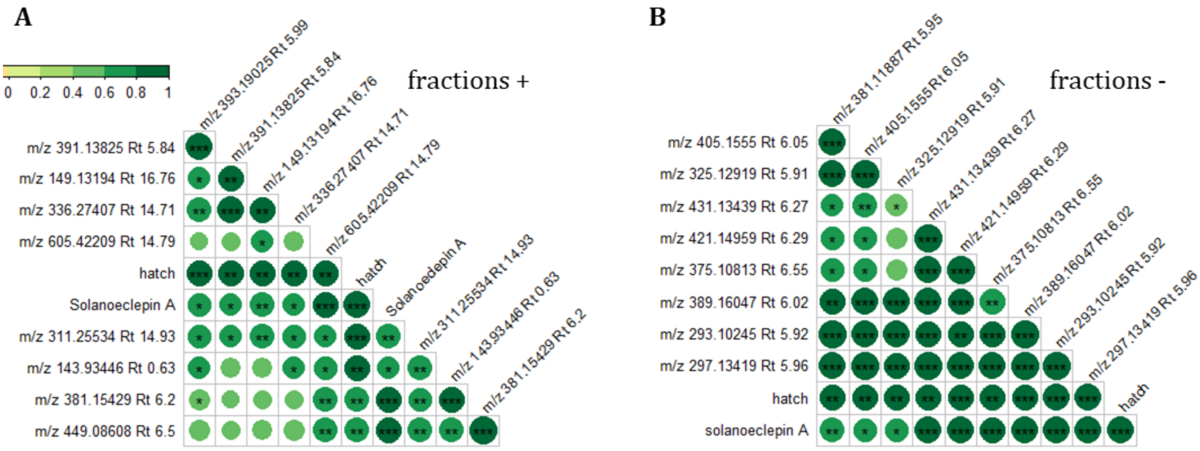

Figure S4: the top 10 Pearson's correlations between metabolites in positive (A) and negative (B) mode detected by LC-ESI-QTOF-MS analysis of fractions 8, 9, 10 and 11 of Avatar, Desiree and Seresta cultivars and hatching of PCN.

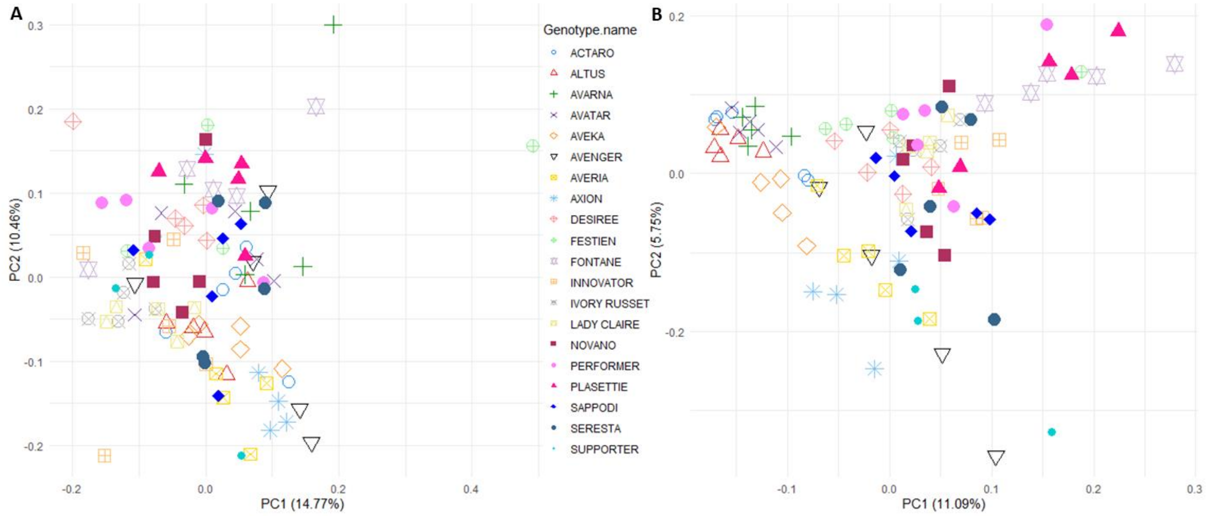

Figure S5: PCA plots of positive (A) and negative (B) mode of LC-ESI-QTOF-MS data from root extract samples of selected genotypes (Figure 1A)

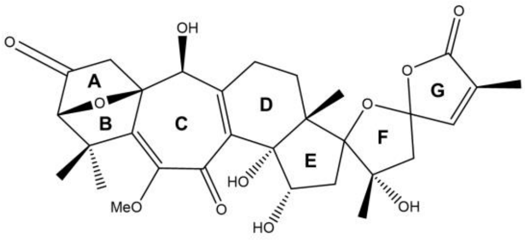

Chemical Formula: $C_{30}H_{36}O_{11}$
Exact Mass: 572.23
Molecular Weight: 572.61

Figure S6: putative structure of compound detected in root extract that highly correlates with solA as well as hatching with m/z 571.22 (-).
